# The analysis of decimation and interpolation in the linear canonical transform domain

**DOI:** 10.1186/s40064-016-3479-4

**Published:** 2016-10-13

**Authors:** Shuiqing Xu, Yi Chai, Youqiang Hu, Lei Huang, Li Feng

**Affiliations:** 1 College of Automation, Chongqing University, Chongqing, China; 2 State Key Laboratory of Power Transmission Equipment and System Security and New Technology, Chongqing University, Chongqing, China

**Keywords:** Linear canonical transform, Decimation and interpolation, Polyphase network, Differential filter

## Abstract

Decimation and interpolation are the two basic building blocks in the multirate digital signal processing systems. As the linear canonical transform (LCT) has been shown to be a powerful tool for optics and signal processing, it is worthwhile and interesting to analyze the decimation and interpolation in the LCT domain. In this paper, the definition of equivalent filter in the LCT domain have been given at first. Then, by applying the definition, the direct implementation structure and polyphase networks for decimator and interpolator in the LCT domain have been proposed. Finally, the perfect reconstruction expressions for differential filters in the LCT domain have been presented as an application. The proposed theorems in this study are the bases for generalizations of the multirate signal processing in the LCT domain, which can advance the filter banks theorems in the LCT domain.

## Background

The linear canonical transform (LCT) (Bodenheimer et al. 1971; Sheridan 2016; Pei and Ding 2002; Xu et al. 2015b, a), which was introduced in the 1970s, is an integral transform with three free parameters. Many widely used linear transform in optical system modeling and digital signal processing, such as the Fourier transform (FT), the fractional Fourier transform (FrFT), the Fresnel transform and scaling operations are all special cases of the LCT (Xu et al. 2015b, a; Almeida 1994; Sharma et al. 2013; Zhao et al. 2014; Shi et al. 2014). Due to its extra degrees of freedom, the LCT is more flexible and has been shown to be a powerful tool for optics, filter design, signal synthesis, time-frequency analysis, phase retrieval, pattern recognition, encryption, modulation, multiplexing in communication and many other areas (Zhang 2016b; Zhao et al. 2010, 2009; Song and Zhao 2014; Zhang 2015; Sharma and Joshi 2006; Zhang 2016a; Stern 2008; Li et al. 2007). Therefore, developing the relevant theorems for LCT are of importance in optical systems and many signal processing applications.

Simultaneously, computational amount and storage load have gradually increased due to the rapid development of digital signal processing. In order to decrease the computational amount and storage load, different sampling rates and the conversion between them are typically required in many real applications, such as image processing, digital audio and communications. Under these circumstances, the theory of multirate signal processing was introduced and improved in Vaidyanathan (1990). Decimation and interpolation are the two basic building blocks in the multirate digital signal processing systems. The decimator is utilized to decrease the sampling rate and interpolator to increase the sampling rate. What’s more, the FT and FrFT domain analysis of decimation and interpolation in the multirate digital signal processing have been well studied in Vaidyanathan (1990), Tao et al. (2008), Meng et al. (2007). As the LCT has recently been found many applications in optics and digital signal processing, the relevant theorems, such as convolution theorems, uncertainty principle theorems, sampling theorems, and others in the LCT domain have been well established (Zhang 2016b; Zhao et al. 2009, 2010; Song and Zhao 2014; Zhang 2015; Sharma and Joshi 2006; Zhang 2016a). However, to the best of our knowledge, the analysis of decimation and interpolation in the LCT domain has never been presented before. It is therefore theoretically interesting and practically useful to analyze the decimation and interpolation in the LCT domain.

Our objective in the paper is to study the LCT domain analysis of decimation and interpolation, which can not only generalize the relevant theories of the FT and the FRFT, but also act as the basis of multirate signal processing theorems in the LCT domain. We firstly give the definition of equivalent filter in the LCT domain, which is a generalization of the equivalent filter in the FRFT domain. Then, due to the polyphase decomposition is very fundamental to the efficient implementation of decimation and interpolation in the multirate digital signal processing systems, the polyphase networks for decimator and interpolator in the LCT domain have been proposed based on the definition of equivalent filter. Finally, as an application, the perfect reconstruction expressions for differential filters in the LCT domain have been presented.

## Methods

### The linear canonical transform 

The continuous-time LCT with parameter $A=(a,b,c,d)$ of a signal or function *x*(*t*) denoted by $L_x^A (u)$, is defined as Bodenheimer et al. (1971)

1 $$\begin{aligned} X_A (u)&= L_x^A (u) = L_A [x(t)](u) = \left\{ \begin{array}{ll} \int _{ - \infty }^\infty {x(t)K_A (u,t)} dt,&{} \quad b \ne 0 \\ \sqrt{d} e^{j(cd/2)u^2 } x(du), &{} \quad b = 0 \\ \end{array} \right. \end{aligned}$$

where *a*, *b*, *c*, *d* are real numbers satisfying $ad-bc=1$, and the kernel $K_A (u,t)$ is given by

2 $$\begin{aligned} K_A (u,t) = C_A e^{j\left(\frac{a}{{2b}}t^2 - \frac{1}{b}tu + \frac{d}{{2b}}u^2 \right)} \end{aligned}$$

and $C_A = \sqrt{1/j2\pi b}$. It is noted that when $b=0$, the LCT of a signal is essentially a chirp multiplication. So we shall confine our attention to LCT for $b \ne 0$ in the following sections. Conversely, the inverse LCT is expressed as

3 $$\begin{aligned} x(t) = \int _{ - \infty }^\infty {L_x^A (u)K_{A^{ - 1} } } (u,t)du\mathrm{{ }} \end{aligned}$$

where $$A^{ - 1} = (d, - b, - c,a)$$.

### Discrete-time linear canonical transform

In digital signal processing systems, the signals used are digital signals sampled from the analog signal. Their representations in LCT domain should be obtained by discrete-time LCT (DTLCT). The definition of DTLCT have been proposed in Pei and Ding (2000), Koc et al. (2008), Oktem and Ozaktas (2009), Hennelly and Sheridan (2005), Healy and Sheridan (2009) from different perspectives. In this subsection, we introduce the definition in Pei and Ding (2000), i.e. the DTLCT of $x(n) = x_s (t)$ is defined as

4 $$\begin{aligned} {\tilde{X}}_A (u) = \sqrt{\frac{1}{{j2\pi b}}} e^{j(d/2b)u^2 } \sum \limits _{n = - \infty }^{n = \infty } {x(n) e^{j(a/2b)n^2 \Delta t^2 } e^{ - j(1/b)un\Delta t} } \end{aligned}$$

In order to make further studies, the digital frequency in the LCT domain is defined as $w = u \cdot \Delta t$. Substituting *w* into Eq. (4), we have

5 $$\begin{aligned} {\tilde{X}}_A (w) = \sqrt{\frac{1}{{j2\pi b}}} e^{j(d/2b)(w/\Delta t)^2 } \sum \limits _{n = - \infty }^{n = \infty } {x(n)e^{j(a/2b)n^2 \Delta t^2 } e^{ - j(1/b)nw} } \end{aligned}$$

The digital frequency in LCT domain is the instrument in the study of LCT domain analysis of the sampling rate conversion, as well as signal polyphase representation and filter bank theorems in LCT domain.

### Simplified linear canonical transform

The simplified LCT, which has the same capabilities as the original LCT for the design of the LCT filter, digital computation, optical implementation and gradient-index medium system, is defined as Pei and Ding (2000)

6 $$\begin{aligned} \bar{X}_{(a,b,c,d)} (u) = \sqrt{\frac{1}{{j2\pi b}}} \int _{ - \infty }^\infty {x(t)} e^{j(a/2b)t^2 } e^{ - j(1/b)ut} dt \end{aligned}$$

Simultaneously, the inverse simplified LCT is expression as

7 $$\begin{aligned} x(t) = \sqrt{\frac{1}{{ - j2\pi b}}} \int _{ - \infty }^\infty {\bar{X}_{(a,b,c,d)} (u)} e^{j( - a/2b)t^2 } e^{j(1/b)ut} du \end{aligned}$$

## Results

### The LCT domain analysis of decimation and interpolation

To study the decimation and interpolation in the LCT domain, in this section, we firstly give the definition of equivalent filter in the LCT domain. Then, the direct implementation structure for decimation and interpolation in the LCT domain are derived based on the definition. Moreover, the polyphase networks for decimation and interpolation in the LCT domain are also deduced.

#### Equivalent FIR filter in the LCT domain

The convolution theorem for signals in the LCT domain have been introduced in Wei et al. (2012), Deng et al. (2006). Analogously, based on the definition of DTLCT, the convolution theorem in the DTLCT domain can be expressed as:

8 $$\begin{aligned} f(nT)\,=\, & {} \sqrt{\frac{1}{{j2\pi b}}} e^{ - j\frac{a}{{2b}}(nT)^2 } \left[ \left( h(nT)e^{j\frac{a}{{2b}}(nT)^2 } \right) *\left( x(nT)e^{j\frac{a}{{2b}}(nT)^2 }\right) \right] \end{aligned}$$

9 $$\begin{aligned} F_A (w)\,=\, & {} e^{ - j\frac{{dw^2 }}{{2bT^2 }}} X_A (w)H_A (w) \end{aligned}$$

where $F_A (w)$, $X_A (w)$, $H_A (w)$ denotes the DTLCT of signal *f*(*t*), *x*(*t*), *h*(*t*), respectively. From Eq. (9), it is easy to know that the convolution theorem for the DTLCT contains an extra chirp factor and hence does not easily implement in the time domain. On the other hand, by multiplying the $e^{ - j\frac{{dw^2 }}{{2bT^2}}}$ to both sides of Eq. (9), we can obtain

10 $$\begin{aligned} F_A (w)e^{ - j\frac{{dw^2 }}{{2bT^2 }}} = X_A (w)e^{ - j\frac{{dw^2 }}{{2bT^2 }}} H_A (w)e^{ - j\frac{{dw^2 }}{{2bT^2 }}} \end{aligned}$$

According the definition of simplified LCT, we get

11 $$\begin{aligned} \bar{Y}_A (w) = \bar{X}_A (w)\bar{H}_A (w) \end{aligned}$$

Equation (11) shows that the convolution of two signals is equivalent to simple multiplication of their simplified LCTs in the simplified LCT domain. It is more useful in practical filtering. Based on this, we give the definition of the equivalent filter in the LCT domain as follows.

##### **Definition 1**

Suppose $H_A (w)$ denotes the DTLCT of the finite length sequence *h*(*nT*), then we define $\bar{H}_A (w)$ as the equivalent filter in the LCT domain.

12 $$\begin{aligned} \bar{H}_A (w) = H_A (w)e^{ - j\frac{{dw^2 }}{{2bT^2 }}} \end{aligned}$$

This definition shows that the equivalent FIR filter in the LCT domain does not contain an extra chirp factor and is easy to implement in the time domain. In addition, when substituting special parameters into the equivalent FIR filter for the LCT, the equivalent FIR filter in the FRFT domain can be obtained.

#### The polyphase implementation of decimation and interpolation in the LCT domain

The polyphase decomposition is very fundamental to the efficient implementation of decimation and interpolation in the multirate digital signal processing systems. It can be applied for the derivation of new sampling theorems and the recovering bandlimited signal from nonuniformly sampled versions. Theories and applications of polyphase decomposition for the decimation and interpolation in the FT and FRFT domain have been well studied in Vaidyanathan (1990), Tao et al. (2008), Meng et al. (2007). To obtain the polyphase implementation of decimation and interpolation in the LCT domain, we study the direct implementation structure for decimation and interpolation in the LCT domain at first.

**Fig. 1:**
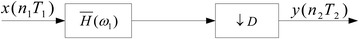
Decimation by the factor *D*

Now, let us consider the definition of equivalent FIR filter, the block diagram notation of decimation process in the LCT domain can be depicted in Fig. 1.

**Fig. 2:**
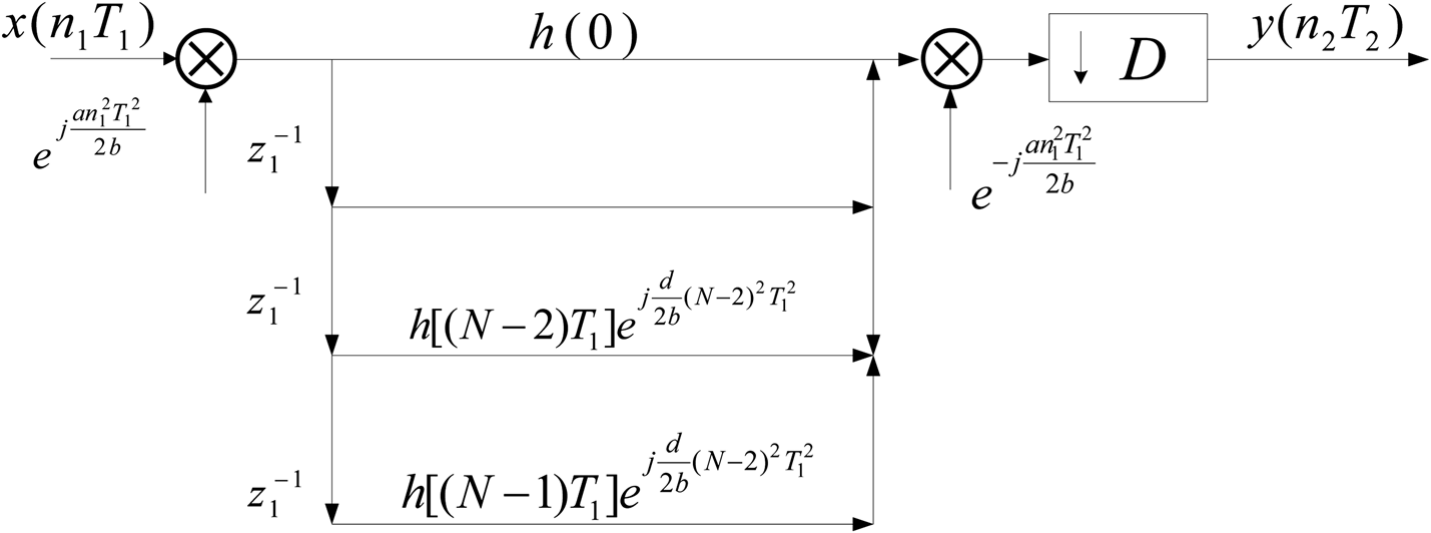
Direct implementation structure of decimator

Then, according to the convolution theorem in the LCT domain, the direct implementation structure for decimation process in the LCT domain can be obtained in Fig. 2.

**Fig. 3:**
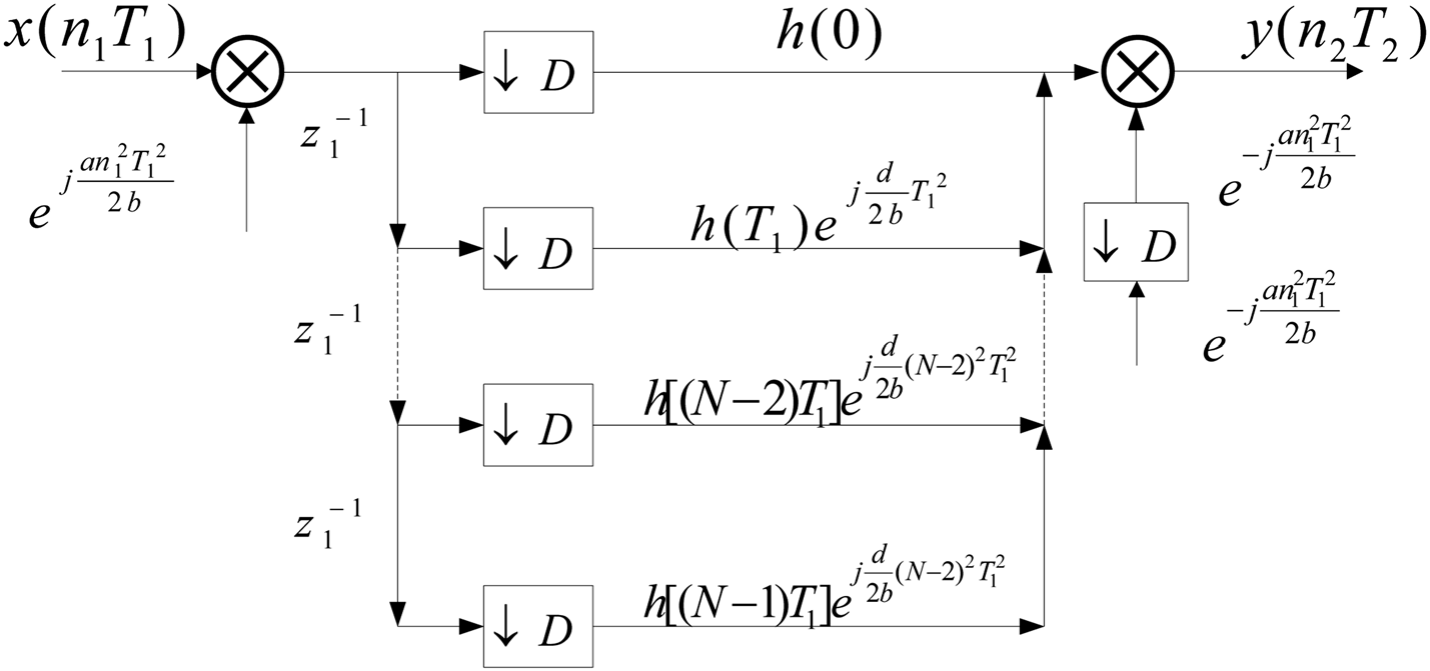
Efficiency implementation structure of decimator

From Fig. 2, it is easy to show that the direct implementation structure for decimation in the LCT domain is inefficient due to every calculation $y(n_1 T_1 )$ needs to be completed within a $T_1$. However, the calculation efficiency can be improved by utilizing the equivalent FIR filter and exchanging the $e^{ - j\frac{{dw^2 }}{{2bT^2 }}}$ and the decimator. Thus, the direct efficiency implementation structure for decimator in LCT domain can be obtained in Fig. 3. It shows that the calculated amount in Fig. 3 reduced to 1 / *D* of the direct implementation structure in Fig. 2. Similar to the decimator case, the efficiency implementation structure for interpolator in the LCT domain also can be obtained.

From above analysis, the direct efficiency implementation structure for decimator and interpolator in the LCT domain have been derived. To obtain the polyphase networks for decimation and implementation in the LCT domain, the polyphase networks for equivalent filter have been derived in the following at first.

Let $\bar{H}_A (w) = H_A (w)e^{ - j\frac{{dw^2 }}{{2bT^2 }}}$ be an equivalent filter, *H*(*w*) is written in the form

13 $$\begin{aligned} H(w) =&\sum \limits _{n = 0}^{Q - 1} {h(nD + 0)e^{j\frac{{dw^2 }}{{2bT^2 }}} e^{j\frac{a}{{2b}}(nD)^2 T^2 } e^{ - j(nD)w/b} } \\&+ \sum \limits _{n = 0}^{Q - 1} {h(nD + 1)e^{j\frac{{dw^2 }}{{2bT^2 }}} e^{j\frac{a}{{2b}}(nD + 1)^2 T^2 } e^{ - j(nD + 1)w/b} } \\&+ \cdots \\&+ \sum \limits _{n = 0}^{Q - 1} {h(nD + k)e^{j\frac{{dw^2 }}{{2bT^2 }}} e^{j\frac{a}{{2b}}(nD + k)^2 T^2 } e^{ - j(nD + k)w/b}} \end{aligned}$$

where $N = QD$ stands for the length of FIR filter in the LCT domain, and

14 $$\begin{aligned} &\sum \limits _{n = 0}^{Q - 1} {h(nD + k)e^{j\frac{{dw^2 }}{{2bT^2 }}} e^{j\frac{a}{{2b}}(nD + k)^2 T^2 } e^{ - j(nD + k)w/b} }\\&\quad = e^{ - jkw/b} e^{j\frac{a}{{2b}}k^2 T^2 } e^{j\frac{{dw^2 }}{{2bT^2 }}} \sum \limits _{n = 0}^{Q - 1} {h(nD + k) } \times e^{j\frac{a}{{2b}}(n^2 D^2 + 2knD)T^2 } e^{ - jnDw/b} \end{aligned}$$

Then, suppose

15 $$\begin{aligned} E_k (Dw)&= e^{j\frac{{dw^2 }}{{2bT^2 }}} \sum \limits _{n = 0}^{Q - 1} {\left( h(nDT + kT)e^{j\frac{{2aknDT{}^2}}{{2b}}} \right) } \times e^{j\frac{{an^2 D^2 T{}^2}}{{2b}}} e^{ - jnDw/b} \\&= F_A [g_k (nDT)] \end{aligned}$$

where

16 $$\begin{aligned} g_k (nDT) = h(nDT + kT)e^{j\frac{{2aknDT{}^2}}{{2b}}} \end{aligned}$$

Therefore, we can obtain

17 $$\begin{aligned} H(w) = \sum \limits _{k = 0}^{D - 1} {e^{ - jkw/b} e^{j\frac{a}{{2b}}k^2 T^2 } } E_k (Dw) \end{aligned}$$

According to Eq. (12), we can rewrite Eq. (17) as

18 $$\begin{aligned} \bar{H}(w) = \sum \limits _{k = 0}^{D - 1} {e^{ - jkw/b} e^{j\frac{a}{{2b}}k^2 T^2 } } \bar{E}_k (Dw) \end{aligned}$$

where

19 $$\begin{aligned} \bar{E}_k (Dw) = \sum \limits _{n = 0}^{Q - 1} {\left( h(nDT + kT)e^{j\frac{{2aknDT{}^2}}{{2b}}} \right) e^{j\frac{{an^2 D^2 T{}^2}}{{2b}}} e^{ - jnDw/b} } \end{aligned}$$

On the other hand, from the properties of the LCT, we get

20 $$\begin{aligned} F_A [\delta (nT - kT)]&= K_A e^{j\frac{{dw^2 }}{{2bT^2 }}} \sum \limits _{n = - \infty }^\infty {\delta (nT - kT)e^{j\frac{a}{{2b}}(nT)^2 } e^{ - jnw/b} } \\&= K_A e^{j\frac{{dw^2 }}{{2bT^2 }}} e^{j\frac{a}{{2b}}(kT)^2 } e^{ - jkw/b} \\ \end{aligned}$$

From above all, we obtain

21 $$\begin{aligned} H(w)&= \sum \limits _{k = 0}^{D - 1} {e^{ - jkw/b} e^{j\frac{a}{{2b}}k^2 T^2 } } E_k (Dw) \\&= \frac{1}{{K_A }}e^{ - j\frac{{dw^2 }}{{2bT^2 }}} \sum \limits _{k = 0}^{D - 1} {F_A [\delta (nT - kT)]} E_k (Dw) \\ \end{aligned}$$

Assume that

22 $$\begin{aligned} Y_A (w) = X_A (w)\bar{H}_A (w) \end{aligned}$$

Substituting Eq. (21) into Eq. (22) and rearranging it, we get

23 $$\begin{aligned} Y_A (w)e^{j\frac{{dw^2 }}{{2bT^2 }}}&= X_A (w)H_A (w) \\&= \frac{1}{{K_A }}\sum \limits _{k = 0}^{D - 1} {e^{ - j\frac{{dw^2 }}{{2bT^2 }}} } X_A (w)F_A [\delta (nT - kT)]E_k (Dw) \\ \end{aligned}$$

From the convolution theorem in the LCT domain, $X_A (w)$ can be written as

24 $$\begin{aligned} X_A (w)&= \frac{1}{{K_A }}e^{ - j\frac{{dw^2 }}{{2bT^2 }}} X_A (w)F_A [\delta (nT - kT)] \\&= F_A \left[ e^{ - j\frac{{an^2 T^2 }}{{2b}}} x(nT - kT)e^{j\frac{{a(n - k)^2 T^2 }}{{2b}}} e^{j\frac{{ak^2 T^2 }}{{2b}}} \right] \\ \end{aligned}$$

From Eqs. (23) and (24), we have

25 $$\begin{aligned} Y_A (w)&= e^{ - j\frac{{dw^2 }}{{2bT^2 }}} E_k (Dw) \sum \limits _{k = 0}^{D - 1} {F_A \left[ e^{ - j\frac{{an^2 T^2 }}{{2b}}} x(nT - kT) e^{j\frac{{a(n - k)^2 T^2 }}{{2b}}} e^{j\frac{{ak^2 T^2 }}{{2b}}}\right] } \\&= \sum \limits _{k = 0}^{D - 1} {F_A \left[ e^{ - j\frac{{an^2 T^2 }}{{2b}}} x(nT - kT)e^{j\frac{{a(n - k)^2 T^2 }}{{2b}}} e^{j\frac{{ak^2 T^2 }}{{2b}}}\right] } \bar{E}_k (Dw) \end{aligned}$$

**Fig. 4:**
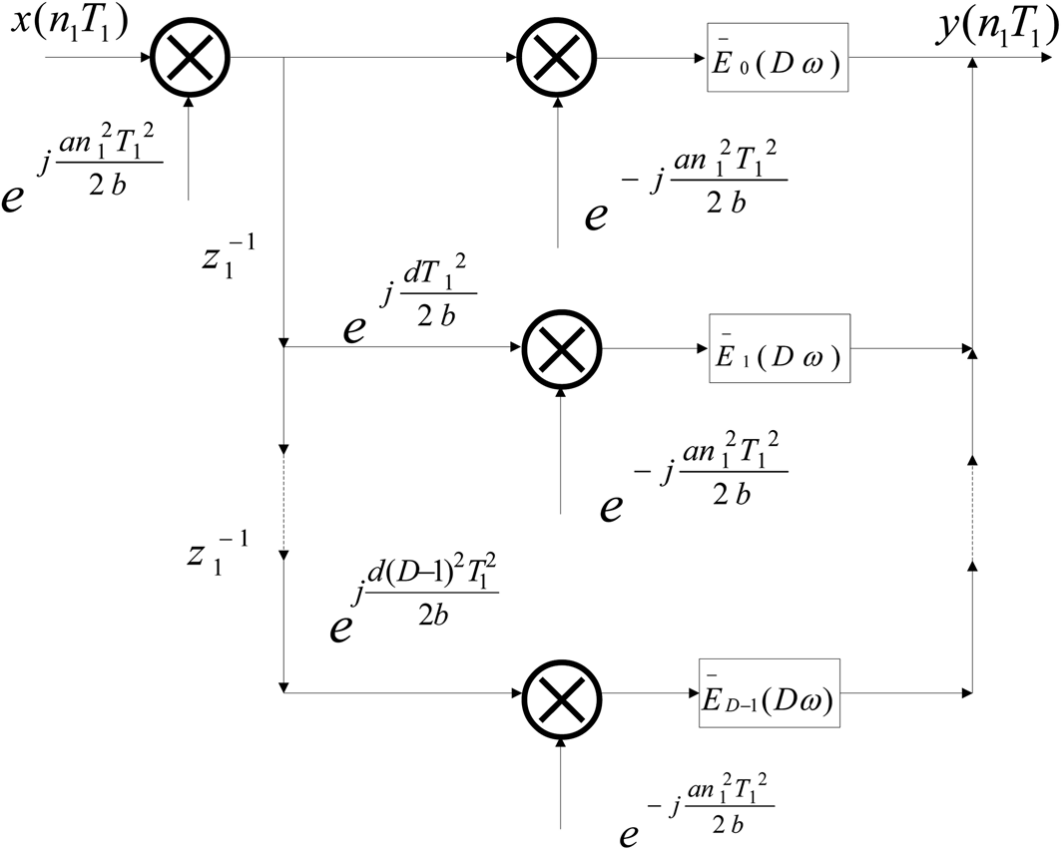
Polyphase implementation of equivalent filter in the LCT domain

Based on this equation, the polyphase implementation of equivalent FIR filter in the LCT domain can be derived in Fig. 4.

**Fig. 5:**
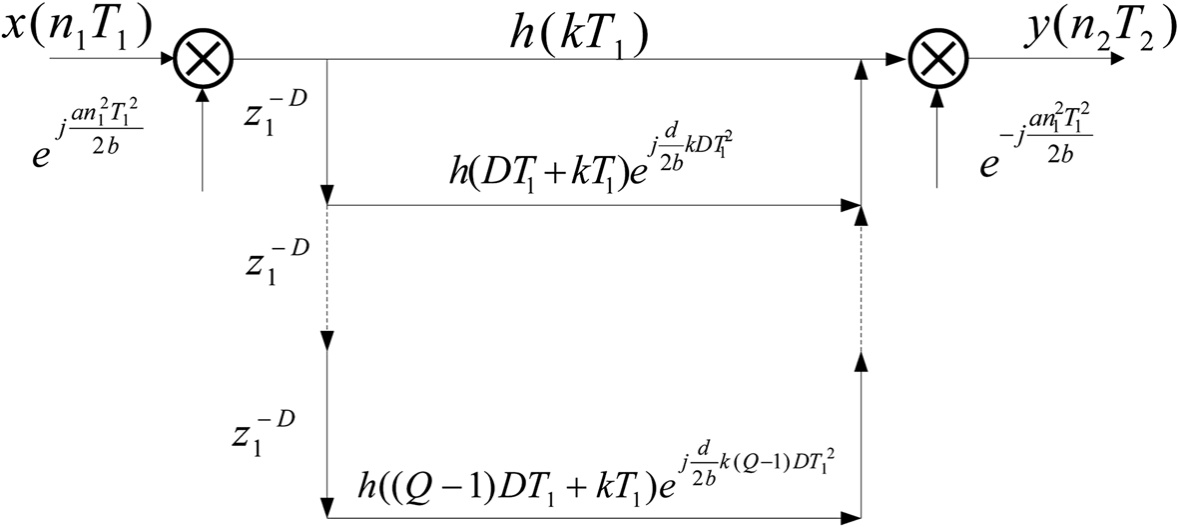
Polyphase implementation of $\bar{E}_k (Dw)$

Similarly, the polyphase implementation of $\bar{E}_k (Dw)$ can be obtained in Fig. 5.

**Fig. 6:**
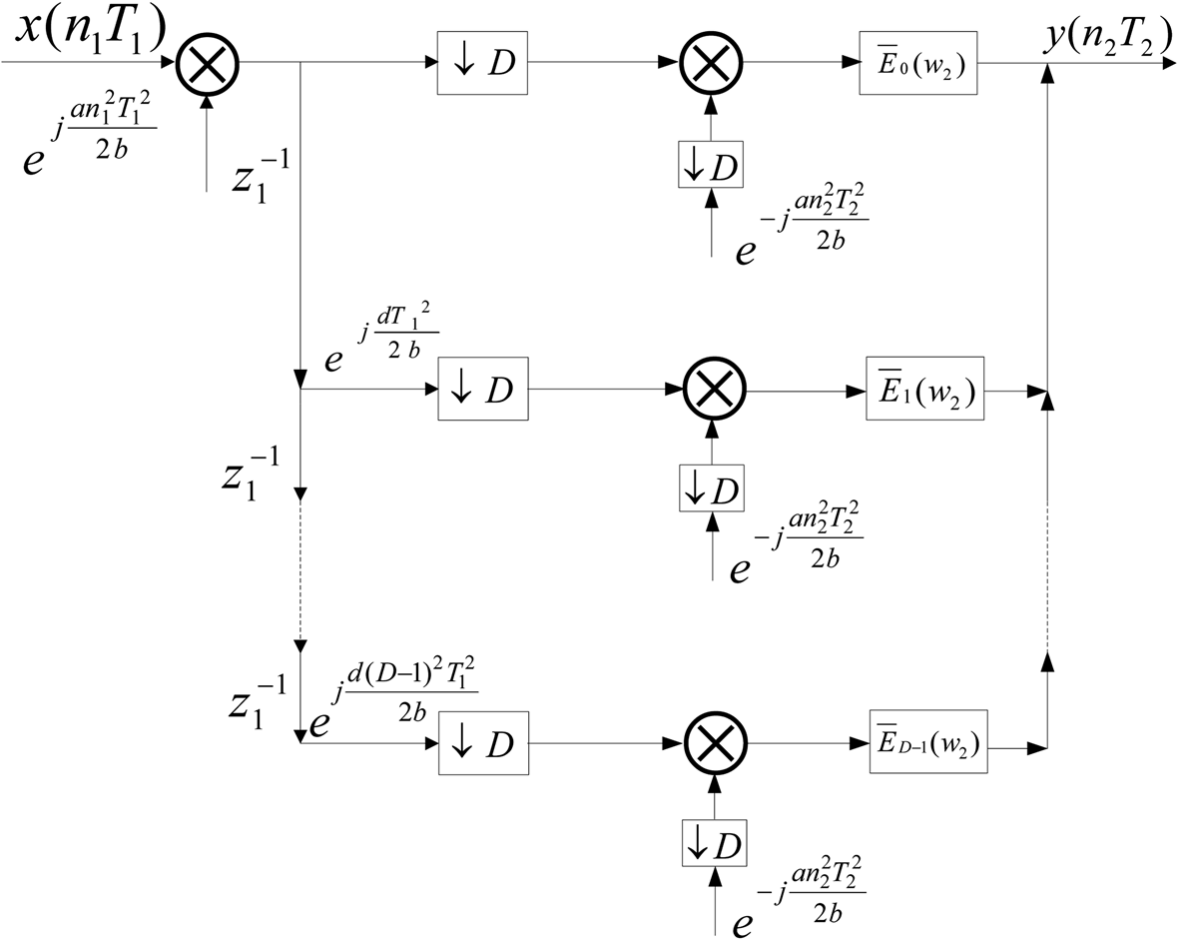
Polyphase implementation of decimator

Then, according to Figs. 1 and 4, the polyphase implementation structure for decimation in LCT domain can be obtained in Fig. 6. Likewise, the polyphase implementation structure for interpolation in LCT domain also can be derived.

Furthermore, the polyphase implementation structure for decimation and interpolation in LCT domain have many real applications, such as it can perform digital filtering the general case, it also can offer significant saving in computation rate and hardware in two important applications, sampling rate alternation and realization of filter banks.

## Discussion

### Application in the LCT differential filter

**Fig. 7:**
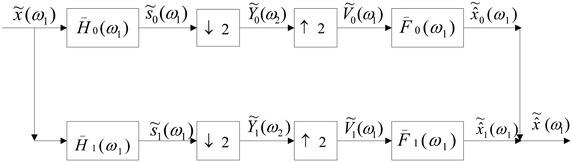
The differential filters in the LCT domain

Differential sampling can be used to decrease the computational amount and storage load in the system. In this section, the application of decimation and interpolation in the LCT differential filter have been studied. Based on the analysis in previous section, the perfect reconstruction expressions for differential filters in the LCT domain can be obtained. Here, the LCT differential filters depicted in Fig. 7 is considered. Following the method for the FRFT, the first order difference in the LCT domain is defined as $x(n_1 T_1 ) - x(n_1 T_1 - T_1 )e^{ - j(n - 1)T_1 ^2 d/b}$. Thus, the output of the system in Fig. 7 can be written as:

26 $$\begin{aligned} \tilde{\hat{X}}_0 (w_1 )&= V_0 (w_1 )\bar{F}_0 (w_1 ) = Y_0 (w_1 )\bar{F}_0 (w_1 ) \\&= \bar{F}_0 (w_1 )\left[ \frac{1}{2}\sum \limits _{k = 0}^1 {X_A } \left( w_1 - \frac{{2k\pi b}}{2}\right) \bar{H}_0 \left( w_1 - \frac{{2k\pi b}}{2}\right) e^{j 2k\pi b \frac{{(w_1 - 2k\pi b/2)}}{{2T_1 ^2 }}} \right] \end{aligned}$$

27 $$\begin{aligned} \tilde{\hat{X}}_1 (w_1 )&= V_1 (w_1 )\bar{F}_1 (w_1 ) = Y_1 (w_1 )\bar{F}_1 (w_1 ) \\&= \bar{F}_1 (w_1 )\left[ \frac{1}{2}\sum \limits _{k = 0}^1 {X_A } \left( w_1 - \frac{{2k\pi b}}{2}\right) \bar{H}_1 \left(w_1 - \frac{{2k\pi b}}{2}\right)e^{j 2k\pi b \frac{{(w_1 - 2k\pi b/2)}}{{2T_1 ^2 }}} \right] \\ \end{aligned}$$

Therefore, the $\tilde{\hat{X}}(w_1 )$ is equal to

28 $$\begin{aligned} \tilde{\hat{X}}(w_1 ) &=\tilde{\hat{X}}_0 (w_1 ) + \tilde{\hat{X}}_1 (w_1 )= V_0 (w_1 )\bar{F}_0 (w_1 ) + V_1 (w_1 )\bar{F}_1 (w_1 ) \\ &=\frac{1}{2}X_A (w_1 )((\bar{F}_0 (w_1 )\bar{H}_0 (w_1 ) + \bar{F}_1 (w_1 )\bar{H}_1 (w_1 )) \\&\quad + \frac{1}{2}X_A (w_1 - \pi b)(\bar{F}_0 (w_1 )\bar{H}_0 (w_1 - \pi b) + \bar{F}_1 (w_1 )\bar{H}_1 (w_1 - \pi b)) e^{j\pi b (w_1 - \pi b)/T_1 ^2 } \end{aligned}$$

On the other hand, from the equivalent filter in the LCT domain, we have

29 $$\begin{aligned} S_0 (w_1 ) = H_0 (w_1 )X_A (w_1 )e^{ - j\frac{{dw_1 ^2 }}{{2bT_1 ^2 }}} \end{aligned}$$

30 $$\begin{aligned} S_1 (w_1 ) = H_1 (w_1 )X_A (w_1 )e^{ - j\frac{{dw_1 ^2 }}{{2bT_1 ^2 }}} \end{aligned}$$

where

31 $$\begin{aligned} H_0 (w_1 )\,=\, & {} L_A [\delta (n_1 T_1 )] \,=\, e^{j\frac{{dw_1 ^2 }}{{2bT_1 ^2 }}} \end{aligned}$$

32 $$\begin{aligned} H_1 (w_1 )\,=\, & {} L_A [\delta (n_1 T_1 ) - \delta (n_1 T_1 - T_1 )] = e^{j\frac{{dw_1 ^2 }}{{2bT_1 ^2 }}} - e^{j\frac{{dw_1 ^2 }}{{2bT_1 ^2 }}} e^{j\frac{{aT_1 ^2 }}{{2b}}} e^{ - jw_1 /b} \end{aligned}$$

From Eqs. (29) to (32), we get

33 $$\begin{aligned} s_0 (n_1 T_1 )&= K_A e^{ - j\frac{a}{{2b}}n_1^2 T_1^2 } \left[x(n_1 T_1 )e^{j\frac{a}{{2b}}n_1^2 T_1^2 } * \delta (n_1 T_1 )e^{j\frac{a}{{2b}}n_1^2 T_1^2 } \right] = x(n_1 T_1 ) \end{aligned}$$

34 $$\begin{aligned} s_1 (n_1 T_1 )&= K_A e^{ - j\frac{a}{{2b}}n_1^2 T_1^2 }\left[ x(n_1 T_1 )e^{j\frac{a}{{2b}}n_1^2 T_1^2 } * (\delta (n_1 T_1 ) - \delta (n_1 T_1 - T_1 ))e^{j\frac{a}{{2b}}n_1^2 T_1^2 } \right] \nonumber \\&= x(n_1 T_1 ) - x(n_1 T_1 - T_1 ))e^{ - j(n - 1)T_1^2 a/b} \end{aligned}$$

From the above equations, we can get the the equivalent filter in the system as

35 $$\begin{aligned} \bar{H}_0 (w_1 )\,=\, & {} H_0 (w_1 )e^{ - j\frac{{dw_1 ^2 }}{{2bT_1 ^2 }}} = 1 \end{aligned}$$

36 $$\begin{aligned} \bar{H}_1 (w_1 )\,=\, & {} H_1 (w_1 )e^{ - j\frac{{dw_1 ^2 }}{{2bT_1 ^2 }}} = 1 - e^{j\frac{{aT_1 ^2 }}{{2b}}} e^{ - jw_1 /b} \end{aligned}$$

It is easy to find that to recovery the original signal in the LCT differential filter, the following equation should be established.

37 $$\begin{aligned} \bar{F}_0 (w_1 )\bar{H}_0 (w_1 ) + \bar{F}_1 (w_1 )\bar{H}_1 (w_1 ) = K \end{aligned}$$

38 $$\begin{aligned} \bar{F}_0 (w_1 )\bar{H}_0 (w_1 - \pi b) + \bar{F}_1 (w_1 )\bar{H}_1 (w_1 - \pi b) = 0 \end{aligned}$$

From Eqs. (35) to (38), we obtain

39 $$\begin{aligned}&F_0 (w_1 )e^{ - j\frac{{dw_1^2 }}{{2bT_1^2 }}} + F_1 (w_1 )e^{ - j\frac{{dw_1^2 }}{{2bT_1^2 }}} \left(1 - e^{j\frac{{aT_1^2 }}{{2b}}} e^{ - jw_1 /b} \right) = K \end{aligned}$$

40 $$\begin{aligned}&F_0 (w_1 )e^{ - j\frac{{dw_1^2 }}{{2bT_1^2 }}} + F_1 (w_1 )e^{ - j\frac{{dw_1^2 }}{{2bT_1^2 }}} \left(1 - e^{j\frac{{aT_1^2 }}{{2b}}} e^{ - j(w_1 - \pi b)/b} \right) = 0 \end{aligned}$$

Then we have

41 $$\begin{aligned} \bar{F}_1 (w_1 )= & {} \frac{K}{{e^{jaT_1^2 /2b} (e^{ - j(w_1 - \pi b)/b} - e^{ - jw_1 /b} )}} \end{aligned}$$

42 $$\begin{aligned} \bar{F}_1 (w_1 )= & {} \frac{{K(e^{jaT_1^2 /2b} e^{ - j(w_1 - \pi b)/b} - 1)}}{{e^{jaT_1^2 /2b} (e^{ - j(w_1 - \pi b)/b} - e^{ - jw_1 /b} )}} \end{aligned}$$

The above two equations are the conditions that the original signal can be perfect reconstruction from differential filters in the LCT domain. It can be used to decrease computational amount and storage load in digital signal processing system. In addition, when substituting special parameters into the LCT differential filters, the perfect reconstruction expressions from differential filters in the FT and FRFT domain can be obtained. Therefore, we conclude that the formula we derived is more general and useful.

## Conclusions

This paper has analyzed the decimation and interpolation in the LCT domain, which can advance the theorems for multirate signal processing in the LCT domain. First, the definition of equivalent FIR filter in the LCT domain has been proposed. By using the definition, the direct implementation structure for decimation and interpolation in the LCT domain have been derived. In addition, the polyphase implementation of decimation and interpolation in the LCT domain also have been obtained. Finally, as an application, the perfect reconstruction expressions for differential filters in the LCT domain have been obtained. The future research and work will be in the direction of real applications of the multirate signal processing theories in the LCT domain.

## References

[CR1] Almeida LB (1994). The fractional Fourier transform and time–frequency representations. IEEE Trans Signal Process.

[CR2] Bodenheimer MM, Banka VS, Helfant RH (1971). Linear canonical transformations and their unitary representations. J Math Phys.

[CR3] Deng B, Tao R, Wang Y (2006). Convolution theorems for the linear canonical transform and their applications. Sci China.

[CR4] Healy JJ, Sheridan JT (2009). Sampling and discretization of the linear canonical transform. Signal Process.

[CR5] Hennelly BM, Sheridan JT (2005). Fast numerical algorithm for the linear canonical transform. J Opt Soc Am A Opt Image Sci Vis.

[CR6] Koc A, Ozaktas HM, Candan C, Alper Kutay M (2008). Digital computation of linear canonical transforms. IEEE Trans Signal Process.

[CR7] Li BZ, Tao R, Wang Y (2007). New sampling formulae related to linear canonical transform. Signal Process.

[CR8] Meng XY, Tao R, Wang Y (2007). Fractional fourier domain analysis of decimation and interpolation. Sci China.

[CR9] Oktem FS, Ozaktas HM (2009). Exact relation between continuous and discrete linear canonical transforms. IEEE Signal Process Lett.

[CR10] Pei SC, Ding JJ (2000). Closed-form discrete fractional and affine fourier transforms. IEEE Trans Signal Process.

[CR11] Pei SC, Ding JJ (2000). Simplified fractional Fourier transforms. J Opt Soc Am A Opt Image Sci Vis.

[CR12] Pei SC, Ding JJ (2002). Eigenfunctions of linear canonical transform. IEEE Trans Signal Process.

[CR13] Sharma KK, Joshi SD (2006). Signal separation using linear canonical and fractional Fourier transforms. Opt Commun.

[CR14] Sharma KK, Sharma L, Sharma S (2013). Paley–wiener criterion in linear canonical transform domains. Signal Image Video Process.

[CR15] Sheridan JHKO (2016). Linear canonical transforms—theory and applications.

[CR16] Shi J, Liu X, Zhang Q, Zhang N (2014). Sampling theorems in function spaces for frames associated with linear canonical transform. Signal Process.

[CR17] Song D, Zhao H (2014). Stochastic formulation of (a, b, c, d)-bandlimited signal reconstruction. Circuits Syst Signal Process.

[CR18] Stern A (2008). Uncertainty principles in linear canonical transform domains and some of their implications in optics. J Opt Soc Am A Opt Image Sci Vis.

[CR19] Tao R, Deng B, Zhang WQ, Wang Y (2008). Sampling and sampling rate conversion of band limited signals in the fractional Fourier transform domain. IEEE Trans Signal Process.

[CR20] Vaidyanathan PP (1990). Multirate digital filters, filter banks, polyphase networks, and applications: a tutorial. Proc IEEE.

[CR21] Wei D, Ran Q, Li Y (2012). A convolution and correlation theorem for the linear canonical transform and its application. Circuits Syst Signal Process.

[CR22] Xu S, Chai Y, Hu Y (2015). Spectral analysis of sampled band-limited signals in the offset linear canonical transform domain. Circuits Syst Signal Process.

[CR23] Xu S, Chai Y, Hu Y, Jiang C, Li Y (2015). Reconstruction of digital spectrum from periodic nonuniformly sampled signals in offset linear canonical transform domain. Opt Commun.

[CR24] Zhang ZC (2015). Unified wigner–ville distribution and ambiguity function in the linear canonical transform domain. Signal Process.

[CR25] Zhang ZC (2016). An approximating interpolation formula for bandlimited signals in the linear canonical transform domain associated with finite nonuniformly spaced samples. Optik-Int J Light Electron Opt.

[CR26] Zhang ZC (2016). New wigner distribution and ambiguity function based on the generalized translation in the linear canonical transform domain. Signal Process.

[CR27] Zhao H, Ran QW, Ma J, Tan LY (2009). On bandlimited signals associated with linear canonical transform. IEEE Signal Process Lett.

[CR28] Zhao H, Ran QW, Tan LY, Ma J (2010). Reconstruction of bandlimited signals in linear canonical transform domain from finite nonuniformly spaced samples. IEEE Signal Process Lett.

[CR29] Zhao H, Wang R, Song D (2014). Recovery of bandlimited signals in linear canonical transform domain from noisy samples. Circuits Syst Signal Process.

